# Increased Long-Term Risks of Occupational Diseases in Homecare Nurses: A Nationwide Population-Based Retrospective Cohort Study

**DOI:** 10.1089/whr.2019.0018

**Published:** 2020-08-17

**Authors:** Hua-Yin Hsu, Chia-Chi Hsieh, Yi-Chen Tseng, Chiu-Hsia Hung, Kow-Tong Chen, Chun-Hsiang Wang, Yuan-Tsung Tseng

**Affiliations:** ^1^ Department of Nursing, Tainan Municipal Hospital (Managed by Show Chwan Medical Care Corporation), Tainan, Taiwan (R.O.C.).; ^2^ Department of Nursing, Chang Bing Show-Chwan Memorial Hospital, Changhua, Taiwan (R.O.C.).; ^3^ Department of Obstetrics and Gynecology, China Medical University, An Nan Hospital, Tainan, Taiwan (R.O.C.).; ^4^ Department of Public Health, College of Medicine, National Cheng Kung University, Tainan, Taiwan (R.O.C.).; ^5^ Department of Hepatogastroenterology and Tainan Municipal Hospital (Managed By Show Chwan Medical Care Corporation), Tainan, Taiwan (R.O.C.).; ^6^ Department of Medical Research, Tainan Municipal Hospital (Managed By Show Chwan Medical Care Corporation), Tainan, Taiwan (R.O.C.).

**Keywords:** homecare, nursing institution, occupational diseases

## Abstract

***Background:*** The work of homecare nurses is different from that of general hospital nurses; therefore, it is necessary to understand the risks of occupational diseases in homecare nurses.

***Materials and Methods:*** In this retrospective cohort research conducted from 2000 to 2013, nursing staff comprised the sample obtained from the National Health Insurance Research Database. Nursing staff were subgrouped according to practice site into homecare, medical center, regional hospital, and local community hospital nurses. The control group included 4,108 subjects.

***Results:*** The risk of severe kidney disease was higher in homecare nurses than in medical center nurses (hazard ratio [HR]: 7.3, 95% confidence interval [CI]: 2.45–21.78) and regional hospital nurses (HR: 3.30, 95% CI: 1.37–7.96). The risk of severe liver disease was higher in homecare nurses than in medical center nurses (HR: 1.92, 95% CI: 1.10–3.35) and regional hospital nurses (HR: 2.06, 95% CI: 1.17–3.62).

***Conclusions:*** The prevalence of occupational diseases was higher in homecare nurses than in noncaregivers. The correlation between different practice environments and disease prevalence rates revealed that various types of nurses can be ranked in the following order based on the prevalence of the aforementioned diseases: homecare nurses > local community hospital nurses > regional hospital nurses > medical center nurses.

## Introduction

Given the uniqueness of the work of nursing staff and their unique work conditions, such as 24-hour shifts, nurses are often expected to play the roles of therapists, caregivers, and educators. Due to patients' increasing demands for medical service quality and high nursing workload, nursing staff have become a population with high work fatigue.^1,2^ Studies have shown that many nurses report high work stress.^3^ Shift work causes sleep problems, which result from psychological, physiological, and behavioral deviations caused by negative emotions.^4^ Mental exhaustion, inability to concentrate, work inefficiency, and low resilience result in not only poor quality of care but also attrition among nurses.^5^

Studies have also found that muscular problems are directly or indirectly proportional to the health of caregivers. The aforementioned factors may cause occupational diseases among nurses.^6^ In many studies, the prevalence of several diseases, including musculoskeletal diseases, genitourinary diseases, insomnia, migraine, common diseases among women, menstrual abnormalities, cancer,^7–14^ peptic ulcer problems,^15^ and occupational tuberculosis,^16^ is much higher in caregivers than in noncaregivers due to the work characteristics of caregivers.

Infection through needle contact is a potential occupational injury in homecare nurses.^17^ Homecare nurses differ from general hospital nurses, and their role is more diverse than that of general hospital nurses. Providing homecare is complicated and stressful,^18^ and the additional stress of this occupation may contribute to a considerable burden of chronic disease. As the elderly population increases rapidly, the prevalence of accompanying chronic diseases and dysfunctions also increases,^19^ which, in turn, will consume a large amount of medical and care resources in the long term, leading to the increasing demand for long-term care and highlighting the role of homecare nurses in long-term care.

Past research has indicated that general hospital nurses often develop diseases, but scant research on homecare nurses providing long-term care has been conducted.

This study explored whether homecare nurses are at a high risk of a particular disease. The prevalence of various diseases, including musculoskeletal diseases, menstrual abnormalities, insomnia, and migraine, was determined for homecare nurses relative to nurses working in medical institutions, medical hospitals, regional hospitals, and local community hospitals.

## Materials and Methods

### Data source

The study sample contained all medical claims data of 2 821 069 participants randomly sampled from the National Health Insurance Research Database (NHIRD), and data were evaluated by performing a retrospective cohort analysis. The NHIRD contains the health care data of more than 99% of Taiwan's population of 23.38 million from 1996. The NHIRD compiles general information, including demographic data, clinic visit dates, diseases diagnoses, operation codes, and prescriptions, and has been used widely in academic studies. The study period was from 2000 to 2013. International Classification of Disease, Ninth Revision, Clinical Modification (ICD-9-CM) codes were to identify diseases, and Charlson comorbidity index (CCI) scores^20^ were calculated for the two cohorts. By analyzing cohort models, the disease risk among nurses was determined in comparison with that in the general population. The Institutional Review Board of Show Chwan Memorial Hospital approved this study (IRB no. 1070402) and waived the requirement of informed consent, because the data sets in the NHIRD contain no identifiable personal information.

### Study design

When screening the demographic database, the exclusion criteria were people aged <18 years and male individuals. Second, the cohorts were sampled at a ratio of 1:4 by using the propensity score matching (PSM). The nursing cohort was divided into the subgroups of homecare, medical center, regional hospital, and local community hospital nurses. To ensure that the cohort of the homecare nurses was representative, homecare nurses and controls with at least 6 years of work experience were selected between 2000 and 2013.

**FIG. 1.**
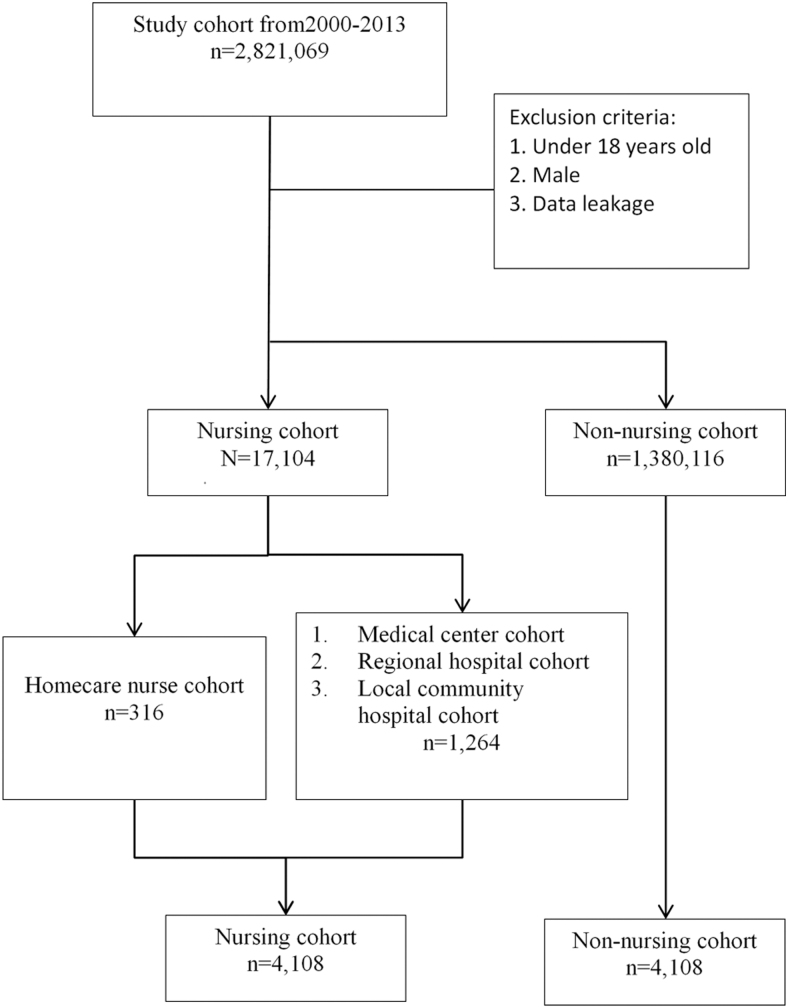
Enrollment of the study sample. Study subjects were identified from a nationwide cohort of 2,821,069 people from 1 January 2000 to 31 December 2013 and were divided into the homecare nurse cohort (*n* = 316) and the comparison group (medical center, regional hospital, and local community nurses, *n* = 1,264) composed of age- and propensity score-matched people.

First, the nursing cohort (*n* = 4,108) and the non-nursing cohort (*n* = 4,108) were matched to analyze the disease prevalence rate in nurses and non-nurses. Second, 316 nurses were included in the homecare nurse cohort, and 1,264 nurses were included in each non-homecare nurse cohort of medical center, regional hospital, and local community hospital nurses, with 3,792 nurses in total (Fig. 1).

### Definitions of variables and study outcome

The longitudinal case–control study was defined by ICD-9-CM. This study estimated the prevalence of high-risk diseases of nurses that have been confirmed in the literature, including myocardial infarction (ICD-9-CM code 410, 412), congestive heart failure (ICD-9-CM code 398.91, 402.01, 402.11, 402.91, 404.01, 404.03, 404.11, 404.13, 404.91, 404.93, 425.4–425.9, 428), peripheral vascular disease (ICD-9-CM code 093.0, 437.3, 440, 441, 443.1–443.9, 447.1, 557.1, 557.9, v43.4), cerebrovascular disease (ICD-9-CM code 362.34, 430–438), dementia (ICD-9-CM code 290, 294.1, 331.2), chronic lung disease (ICD-9-CM code 416.8, 416.9, 490–505, 506.4, 508.1, 508.8), peptic ulcer disease (ICD-9-CM code 531–534), mild liver disease (ICD-9-CM code 070.22, 070.23, 070.32, 070.33, 070.44, 070.54, 070.6, 070.9, 570, 571, 573.3, 573.4, 573.8, 573.9, v42.7), diabetes mellitus (ICD-9-CM code 250), hemiplegia or paraplegia (ICD-9-CM code 334.1, 342, 343, 344.0–344.6, 344.9), renal disease (ICD-9-CM code 403.01, 403.11, 403.91, 404.02, 404.03, 404.12, 404.13, 404.92, 404.93, 582, 583.0–583.7, 585, 586, 588.0, v42.0, v45.1, v56), cancer (ICD-9-CM code 140–172, 174–195.8, 200–208, 238.6), severe liver disease (ICD-9-CM code 456.0–456.2, 572.2–572.8), metastatic carcinoma (ICD-9-CM code 196–199), disk displacement (ICD-9-CM code 722), low back pain (ICD-9-CM code 724.2), back pain (ICD-9-CM code 724.5), peptic ulcer (ICD-9-CM code 531–534), menstrual abnormalities (ICD-9-CM codes 625.3, 625.4, and 626), migraine (ICD-9-CM code 346), sleep disorders (ICD-9-CM code 780.5), and tuberculosis (ICD-9-CM codes 010–018). Excluding the latent observation period of the first year of the disease, this study investigated the disease risk ratio between homecare nurses and non-homecare nurses.

### Statistical analysis

In this study, the paired *t* test, McNemar test, Kaplan–Meier curves, and Cox regression were mainly applied to obtain statistical values and risk assessment statistics, such as hazard ratios (HRs). The matching method applied in this study was PSM, which is the most commonly used method in current big data research. The PSM can effectively reduce the impact of confounding factors. By using the observed baseline covariates, the homecare group was assigned through PSM; the distribution of baseline covariates was similar between the homecare and comparison groups. This could have weakened the effects of confounding factors, including smoking, alcohol intake, and other lifestyle factors in the PSM. All statistical analyses were executed by using SAS software (version 9.1), R (version 3.1), and SPSS (version 21). All statistical results were interpreted as statistically significant differences with a two-tailed *p*-value of <0.05.

## Results

### Disease prevalence rate of nurses and non-nurses

The disease prevalence rate is provided in Table 1. The results showed that nurses (13.4%) had a statistically significant higher prevalence of chronic lung disease than non-nurses (7.4%; *p* < 0.001). The prevalence of connective tissue disease was higher among nurses (4.1%) than among non-nurses (3.0%; *p* = 0.006). The prevalence of peptic ulcer disease was higher among nurses (24.1%) than among non-nurses (19.0%) (*p* < 0.001). The prevalence of mild liver disease was higher among nurses (16.0%) than among non-nurses (9.7%) (*p* < 0.001). The prevalence of severe liver disease was higher among nurses (6.7%) than among non-nurses (3.6%; *p* < 0.001). The prevalence of disk displacement was higher among nurses (11.6%) than among non-nurses (7.8%; *p* < 0.001). The prevalence of low back pain was lower among nurses (7.9%) than among non-nurses (8.4%; *p* = 0.005). The prevalence of back pain was the same (4.9%) among nurses and non-nurses (*p* = 0.008). The prevalence of menstrual abnormalities was higher among nurses (18.1%) than among non-nurses (17.8%; *p* = 0.001). The prevalence of migraine was higher among nurses (4.6%) than among non-nurses (3.2%; *p* < 0.001). The prevalence of tuberculosis was higher among nurses (0.7%) than among non-nurses (0.3%; *p* < 0.001). The prevalence of sleep disorders was higher among nurses (17.0%) than among non-nurses (16.4%; *p* < 0.001).

**Table 1.** Baseline Demographic and Clinical Characteristics of Study Patients

	Homecare^a^ (n = 316)	Center (n = 1,264)	Regional (n = 1,264)	Local (n = 1,264)	Nursing cohort (n = 4,108)	Non-nursing cohort (n = 4,108)
CCI	1.28 ± 1.75	0.84 ± 1.43^***^	1.08 ± 1.49^*^	1.08 ± 1.49^*^	1.02 ± 1.50	0.73 ± 1.36^***^
Age, 21–30 years	54 (17.1)	216 (17.1)	216 (17.1)	216 (17.1)	702 (17.1)	702 (17.1)
Age, 31–40 years	106 (33.5)	424 (33.5)	424 (33.5)	424 (33.5)	1378 (33.5)	1378 (33.5)
Age, 41–50 years	135 (42.7)	540 (42.7)	540 (42.7)	540 (42.7)	1755 (42.7)	1755 (42.7)
Age, >51 years	21 (6.6)	84 (6.6)	84 (6.6)	84 (6.6)	273 (6.6)	273 (6.6)
Myocardial infarction	2 (0.6)	1 (0.1)^*^	1 (0.1)^*^	1 (0.1)^*^	5 (0.1)	8 (0.2)
Congestive heart failure	11 (3.5)	24 (1.9)	46 (3.6)	50 (4)	131 (3.2)	138 (3.4)
Peripheral vascular disease	4 (1.3)	12 (0.9)	19 (1.5)	17 (1.3)	52 (1.3)	45 (1.1)
Cerebral vascular disease	5 (1.6)	7 (0.6)	10 (0.8)	10 (0.8)	32 (0.8)	24 (0.6)
Dementia	2 (0.6)	5 (0.4)	0 (0)	1 (0.1)	8 (0.2)	13 (0.3)
Chronic lung disease	54 (17.1)	127 (10.0)^***^	191 (15.1)	179 (14.2)	551 (13.4)	306 (7.4)^***^
Connective tissue disease	16 (5.1)	63 (5.0)	50 (4.0)	41 (3.2)	170 (4.1)	124 (3)^*^
Peptic ulcer disease	82 (25.9)	235 (18.6)^**^	354 (28)	321 (25.4)	992 (24.1)	779 (19)^***^
Mild liver disease	72 (22.8)	173 (13.7)^***^	197 (15.6)^**^	215 (17.0)^*^	657 (16)	400 (9.7)^***^
Diabetes mellitus	21 (6.6)	51 (4.0)^*^	78 (6.2)	70 (5.5)	220 (5.4)	188 (4.6)
Hemiplegia or paraplegia	4 (1.3)	8 (0.6)	12 (0.9)	22 (1.7)	46 (1.1)	31 (0.8)
Renal disease	11 (3.5)	9 (0.7)^**^	21 (1.7)^*^	27 (2.1)	68 (1.7)	62 (1.5)
Cancer	3 (0.9)	45 (3.6)^*^	51 (4.0)^*^	39 (3.1)^*^	138 (3.4)	129 (3.1)
Leukemia	0 (0)	2 (0.2)	2 (0.2)	1 (0.1)	5 (0.1)	3 (0.1)
Lymphoma	0 (0)	2 (0.2)	0 (0)	2 (0.2)	4 (0.1)	6 (0.1)
Severe liver disease	33 (10.4)	74 (5.9)^**^	77 (6.1)^*^	93 (7.4)	277 (6.7)	149 (3.6)^***^
Metastatic carcinoma	0 (0)	1 (0.1)	2 (0.2)	0 (0)	3 (0.1)	10 (0.2)
Intervertebral disk disorders	43 (13.6)	106 (8.4)^*^	162 (12.8)	166 (13.1)	477 (11.6)	320 (7.8)^***^
Lumbago	30 (9.5)	67 (5.3)^***^	118 (9.3)	109 (8.6)	324 (7.9)	346 (8.4)^***^
Backache	22 (7.0)	37 (2.9)^**^	56 (4.4)^*^	85 (6.7)	200 (4.9)	202 (4.9)
Menstruation disorders	61 (19.3)	230 (18.2)^*^	220 (17.4)^*^	231 (18.3)	742 (18.1)	731 (17.8)^***^
Migraine	21 (6.6)	47 (3.7)^**^	65 (5.1)	55 (4.4)	188 (4.6)	130 (3.2)^***^
Tuberculosis	4 (1.3)	8 (0.6)^*^	12 (0.9)	6 (0.5)^*^	30 (0.7)	11 (0.3)^***^
Sleep disturbances	58 (18.4)	184 (14.6)^***^	212 (16.8)	243 (19.2)	697 (17)	675 (16.4)^***^

Paired *t* test and McNemar test were used for analysis.

^a^ This table provides the descriptive statistics of our study participants from 2000 to 2013. Among them, 4180 were in the nursing cohort and 4108 were in the non-nursing 4,108. We then stratified the nursing cohort by institution at a ratio of 1:4 (*i.e.*, homecare vs. center, homecare vs. regional, and homecare vs. local).

^*^ *p* < 0.05; ^**^p < 0.01;^***^p < 0.001.

CCI, Charlson comorbidity index.

### Disease prevalence rate of homecare nurses and general hospital nurses

The disease prevalence rate in homecare nurses (older than 6 years of seniority) and general hospital nurses is provided in Table 1. The results showed that the prevalence of chronic lung disease was 17.1% in homecare nurses, which is higher than that (10.0%) in medical center nurses (*p* = 0.001). The prevalence of ulcerative diseases was higher in homecare nurses (25.9%) than in medical center nurses (18.6%; *p* = 0.002). The prevalence of mild liver disease was 22.8% in homecare nurses in comparison with 13.7% in medical center nurses, 15.6% in regional hospital nurses, and 17.0% in local community hospital nurses (*p* < 0.001, 0.003, and 0.045, respectively). The prevalence of severe kidney disease was higher in homecare nurses (3.5%) than in medical center nurses (0.7%; *p* = 0.001). The prevalence of severe liver disease was 10.4% in homecare nurses in comparison with 5.9% in medical center nurses and 6.1% in regional hospital nurses (*p* = 0.008, 0.022, respectively). The prevalence of disk displacement was higher in homecare nurses (13.6%) than in medical center nurses (8.4%; *p* = 0.017). The prevalence of low back pain was higher in homecare nurses (9.5%) than in medical center nurses (5.3%; *p* < 0.001). The prevalence of backache was higher in homecare nurses (7.0%) than in medical center nurses (2.9%) and regional hospital nurses (4.4%) (*p* = 0.001, 0.037, respectively). The prevalence of menstrual abnormalities was higher in homecare nurses (19.3%) than in medical center nurses (18.2%; *p* = 0.001). The prevalence of migraine was higher in homecare nurses (6.6%) than in medical center nurses (3.7%, *p* = 0.001). The prevalence of tuberculosis was higher in homecare nurses (1.3%) than in medical center nurses (0.6%; *p* = 0.044). The prevalence of sleep disorders was higher in homecare nurses (18.4%) than in medical center nurses (14.6%; *p* < 0.001).

### Comparison of HR between homecare nurses and general hospital nurses

The HR of homecare nurses and general hospital nurses is displayed in Table 2. The results showed a significant HR for chronic lung disease in homecare nurses (HR: 1.84, 95% confidence interval [CI]: 1.12–3.04). The risk of mild liver disease was higher in homecare nurses than in medical center nurses (HR: 2.00, 95% CI: 1.30–3.08), regional hospital nurses (HR: 1.53, 95% CI: 1.01–2.32), and local community nurses (HR: 1.59, 95% CI: 1.05–2.42). The risk of severe kidney disease was higher in homecare nurses than in medical center nurses (HR: 7.3, 95% CI: 2.45–21.7) and regional hospital nurses (HR: 3.30, 95% CI: 1.37–7.96). The risk of severe liver disease was higher in homecare nurses than in medical center nurses (HR: 1.92, 95% CI: 1.10–3.35) and regional hospital nurses (HR: 2.06, 95% CI: 1.17–3.62). The HR of intervertebral disk displacement in homecare nurses was 1.84 (95% CI: 1.19–2.85). The HR of back pain in homecare nurses was 1.86 (95% CI: 1.22–2.86). The HR of back pain in homecare nurses was 2.49 (95% CI: 1.47–4.23). The HR of menstrual abnormalities in homecare nurses was 1.33 (95% CI: 1.00–1.76). Compared with medical center nurses, the HR of migraine in homecare nurses was 1.86 (95% CI: 1.11–3.12). The HR of sleep disorder in homecare nurses was 1.46 (95% CI: 1.08–1.96).

**Table 2.** Adjusted Hazard Ratio of Different Comorbidities by Medical Institutions

	Homecare vs. center		Homecare vs. regional		Homecare vs. local	
	Case/control	HR (95% CI)	Case/control	HR (95% CI)	Case/control	HR (95% CI)
Chronic lung disease	22/51	1.84 (1.12–3.04)^*^	22/80	1.11 (0.69–1.79)	22/75	1.21 (0.75–1.95)
Peptic ulcer disease	36/117	1.37 (0.94–1.99)	36/171	0.86 (0.60–1.23)	36/145	1.02 (0.71–1.48)
Mild liver disease	30/65	2.00 (1.30–3.08)^**^	30/85	1.53 (1.01–2.32)^*^	30/79	1.59 (1.05–2.42)^*^
Renal disease	9/5	7.3 (2.45–21.78)^***^	9/11	3.30 (1.37–7.96)^**^	9/17	2.13 (0.95–4.77)
severe liver disease	18/39	1.92 (1.10–3.35)^*^	18/36	2.06 (1.17–3.62)^*^	18/46	1.60 (0.93–2.76)
Intervertebral disk disorders	29/65	1.84 (1.19–2.85)^**^	29/111	1.04 (0.69–1.57)	29/107	1.08 (0.72–1.63)
Lumbago	30/70	1.86 (1.22–2.86)^**^	30/116	1.06 (0.71–1.58)	30/108	1.13 (0.76–1.7)
Backache	22/37	2.49 (1.47–4.23)^**^	22/56	1.62 (0.99–2.65)	22/86	1.04 (0.65–1.67)
Menstruation disorders	61/227	1.33 (1.00–1.76)^*^	61/225	1.10 (0.83–1.46)	61/229	1.03 (0.78–1.37)
Migraine	21/47	1.86 (1.11–3.12)^*^	21/65	1.32 (0.81–2.16)	21/57	1.50 (0.91–2.48)
Tuberculosis	4/8	2.02 (0.61–6.71)	4/11	1.46 (0.47–4.59)	4/6	2.70 (0.76–9.57)
Sleep disturbances	58/181	1.46 (1.08–1.96)^*^	58/211	1.14 (0.85–1.52)	58/244	0.98 (0.73–1.30)

Cox regression analysis among nurses working in different medical institutions (adjusted for age and index date) was used for analysis.

^*^ *p* < 0.05; ^**^p < 0.01;^***^p < 0.001.

CI, confidence interval; HR, hazard ratio.

### Comparison of the risks of diseases across different age groups

The HRs of homecare nurses and general hospital nurses across different age groups are shown in Tables 3 and 4. The results showed a significant HR for chronic lung disease in 31–40-year-old homecare nurses compared with similarly aged medical center nurses (HR: 2.42, 95% CI: 1.11–5.29). The risk of mild liver disease was higher in 21–30-year-old homecare nurses than in similarly aged medical center nurses (HR: 13.83, 95% CI: 1.44–20+), in 31–40-year-old homecare nurses than in similarly aged medical center nurses (HR: 2.5, 95% CI: 1.16–5.43), and in 41–50-year-old homecare nurses relative to similarly aged medical center nurses (HR: 2.24, 95% CI: 1.17–4.28), relative to regional hospital nurses (HR: 2.4, 95% CI: 1.25–4.61), and relative to local community hospital nurses (HR: 2.26, 95% CI: 1.18–4.33). The risk of severe kidney disease was higher in 41–50-year-old homecare nurses than in similarly aged medical center nurses (HR: 6.07, 95% CI: 1.01–36.3) and in 51-year-old or older homecare nurses than in similarly aged medical center nurses (HR: 5.43, 95% CI: 1.21–24.25). The risk of severe liver disease was higher in homecare nurses older than 51 years of age than in similarly aged medical center nurses (HR: 3.45, 95% CI: 1.05–11.31). The risk of intervertebral disk displacement was higher in 31–40-year-old homecare nurses than in similarly aged medical center nurses (HR: 2.34, 95% CI: 1.19–4.59), relative to local community nurses (HR: 2.03, 95% CI: 1.05–3.94); 41–50-year-old homecare nurses relative to regional hospital nurses (HR: 0.27, 95% CI: 0.08–0.87), local community nurses (HR: 0.23, 95% CI: 0.07–0.74); and older than 51 years old relative to medical center nurses (HR: 3.88, 95% CI: 1.71–8.81). The risk of low back pain was higher in 31–40-year-old homecare nurses than in similarly aged medical center nurses (HR: 2.43, 95% CI: 1.27–4.65). The risk of back pain was higher in 31–40-year-old homecare nurses than in similarly aged medical center nurses (HR: 2.4, 95% CI: 1.01–5.72) and in older than 51-year-old homecare nurses than in similarly aged medical center nurses (HR: 5.12, 95% CI: 1.37–19.07). The risk of menstrual abnormalities was higher in 31–40-year-old homecare nurses than in similarly aged medical center nurses (HR: 1.69, 95% CI: 1.12–2.54) and regional hospital nurses (HR: 1.52, 95% CI: 1.00–2.31). The risk of migraine was higher in 31–40-year-old homecare nurses than in similarly aged regional hospital nurses (HR: 2.37, 95% CI: 1.05–5.35) and in 51-year-old homecare nurses than in similarly aged medical center nurses (HR: 3.81, 95% CI: 1.28–11.35). The risk of sleep disorders was higher in 31–40-year-old homecare nurses than in similarly aged regional hospital nurses (HR: 1.65, 95% CI: 1.05–2.6) and in older than 51-year-old homecare nurses than in similarly aged medical center nurses (HR: 2.07, 95% CI: 1.12–3.83).

**Table 3.** Adjusted Hazard Ratio Among Nurses Working in Different Medical Institutions Classified by Age

	Age	Homecare vs. center	Homecare vs. regional	Homecare vs. local
		HR (95% CI)	HR (95% CI)	HR (95% CI)
Chronic lung disease	21–30	1.35 (0.14–12.98)	0.82 (0.10–7.01)	20+ (0–20+)
	31–40	2.42 (1.11–5.29)^*^	1.68 (0.80–3.51)	1.31 (0.64–2.67)
	41–50	1.56 (0.66–3.70)	1.01 (0.44–2.31)	1.16 (0.50–2.66)
	>51	1.58 (0.50–4.95)	0.71 (0.24–2.05)	0.92 (0.31–2.71)
Peptic ulcer disease	21–30	1.87 (0.34–10.24)	1.15 (0.23–5.72)	1.82 (0.33–9.96)
	31–40	1.65 (0.85–3.20)	0.91 (0.49–1.71)	1.11 (0.59–2.11)
	41–50	1.72 (0.95–3.12)	1.08 (0.61–1.90)	1.34 (0.75–2.38)
	>51	0.76 (0.34–1.69)	0.53 (0.24–1.17)	0.58 (0.26–1.29)
Mild liver disease	21–30	13.83 (1.44–20+)^*^	3.32 (0.74–14.82)	1.52 (0.40–5.73)
	31–40	2.50 (1.16–5.43)^*^	1.92 (0.92–4.04)	1.61 (0.78–3.32)
	41–50	2.24 (1.17–4.28)^*^	2.40 (1.25–4.61)^**^	2.26 (1.18–4.33)^*^
	>51	0.62 (0.18–2.09)	0.37 (0.11–1.22)	0.66 (0.19–2.23)
Renal disease	21–30	20+ (0–20+)	20+ (0–20+)	20+ (0–20+)
	31–40	20+ (0–20+)	20+ (0–20+)	4.04 (0.25–64.56)
	41–50	6.07 (1.01–36.3)^*^	2.42 (0.58–10.11)	2.42 (0.58–10.14)
	>51	5.43 (1.21–24.25)^*^	2.67 (0.75–9.45)	1.42 (0.45–4.47)
Severe liver disease	21–30	20+ (0–20+)	20+ (0–20+)	0.61 (0.08–4.98)
	31–40	1.81 (0.79–4.17)	2.32 (0.97–5.53)	1.82 (0.79–4.18)
	41–50	1.10 (0.37–3.32)	1.35 (0.43–4.18)	1.24 (0.40–3.80)
	>51	3.45 (1.05–11.31)^*^	2.09 (0.72–6.12)	2.59 (0.85–7.91)

Cox regression analysis among different medical institutions (adjusted for age and index date) was used for analysis.

^*^ *p* < 0.05; ^**^p < 0.01.

**Table 4.** Adjusted Hazard Ratio Between Medical Institutions Classified By Age

	Age	Homecare vs. center	Homecare vs. regional	Homecare vs. local
		HR (95% CI)	HR (95% CI)	HR (95% CI)
Intervertebral disk disorders	21–30	8.16 (0.74–20+)	20 (0–20+)	2.1 (0.38–11.47)
	31–40	2.34 (1.19–4.59)^*^	1.68 (0.89–3.2)	2.03 (1.05–3.94)^*^
	41–50	0.42 (0.13–1.37)	0.27 (0.08–0.87)^*^	0.23 (0.07–0.74)^*^
	>51	3.88 (1.71–8.81)^**^	1.18 (0.60–2.32)	1.61 (0.80–3.25)
Lumbago	21–30	1.35 (0.14–12.98)	1.02 (0.11–9.09)	1.29 (0.13–12.43)
	31–40	2.43 (1.27–4.65)^**^	1.31 (0.72–2.38)	1.44 (0.79–2.65)
	41–50	1.23 (0.53–2.84)	0.79 (0.35–1.76)	0.78 (0.35–1.74)
	>51	2.02 (0.87–4.73)	1.02 (0.46–2.22)	1.15 (0.52–2.54)
Backache	21–30	2.04 (0.37–11.13)	7.88 (0.71–20+)	4.2 (0.59–20+)
	31–40	2.40 (1.01–5.72)^*^	1.88 (0.82–4.32)	0.93 (0.43–2.00)
	41–50	1.95 (0.79–4.78)	2.21 (0.88–5.54)	1.00 (0.44–2.29)
	>51	5.12 (1.37–19.07)^*^	0.81 (0.31–2.13)	0.98 (0.37–2.62)
Menstruation disorders	21–30	1.82 (0.64–5.17)	1.14 (0.41–3.19)	0.41 (0.16–1.06)
	31–40	1.69 (1.12–2.54)^*^	1.52 (1.00–2.31)^*^	1.34 (0.88–2.04)
	41–50	0.93 (0.54–1.60)	0.68 (0.40–1.16)	0.75 (0.44–1.28)
	>51	1.66 (0.83–3.29)	1.30 (0.66–2.55)	1.41 (0.72–2.79)
Migraine	21–30	3.97 (0.25–20+)	0.59 (0.07–4.76)	0.96 (0.11–8.63)
	31–40	1.79 (0.82–3.90)	2.37 (1.05–5.35)^*^	1.59 (0.73–3.43)
	41–50	1.12 (0.42–3.01)	0.67 (0.26–1.74)	1.00 (0.38–2.68)
	>51	3.81 (1.28–11.35)^*^	1.95 (0.74–5.12)	2.59 (0.94–7.14)
Tuberculosis	31–40	1.34 (0.14–12.9)	0.67 (0.08–5.57)	1.00 (0.11–8.98)
	41–50	4.03 (0.57–20+)	1.99 (0.37–10.8)	20.00 (0.0–20.+)
	>51	1.35 (0.14–13+)	4.08 (0.26–20+)	2.04 (0.18–22.4)
Sleep disturbances	21–30	1.89 (0.49–7.30)	1.01 (0.28–3.57)	0.64 (0.19–2.15)
	31–40	1.53 (0.98–2.39)	1.65 (1.05–2.60)^*^	1.08 (0.70–1.66)
	41–50	1.01 (0.57–1.79)	0.82 (0.47–1.46)	0.84 (0.47–1.49)
	>51	2.07 (1.12–3.83)^*^	0.99 (0.56–1.75)	1.09 (0.61–1.93)

Cox regression analysis among nurses working in different medical institutions (adjusted for age and index date) was used for analysis.

^*^ *p* < 0.05; ^**^p < 0.01.

### Comprehensive analysis of disease risks in all institutional nurses

**FIG. 2.**
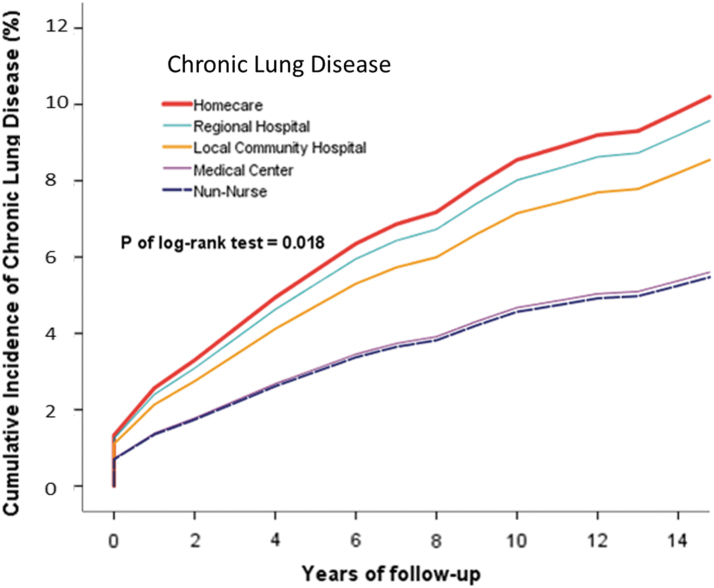
Cumulative incidence of overall chronic lung disease in homecare, medical center, regional hospital, and local community hospital nurse cohorts.

**FIG. 3.**
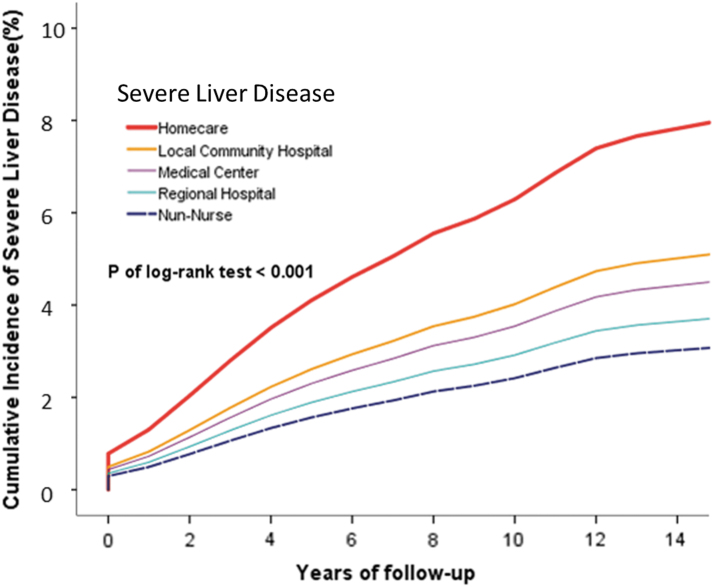
Cumulative incidence of overall severe liver disease between homecare, medical center, regional hospital, and local community hospital nurse cohorts.

**FIG. 4.**
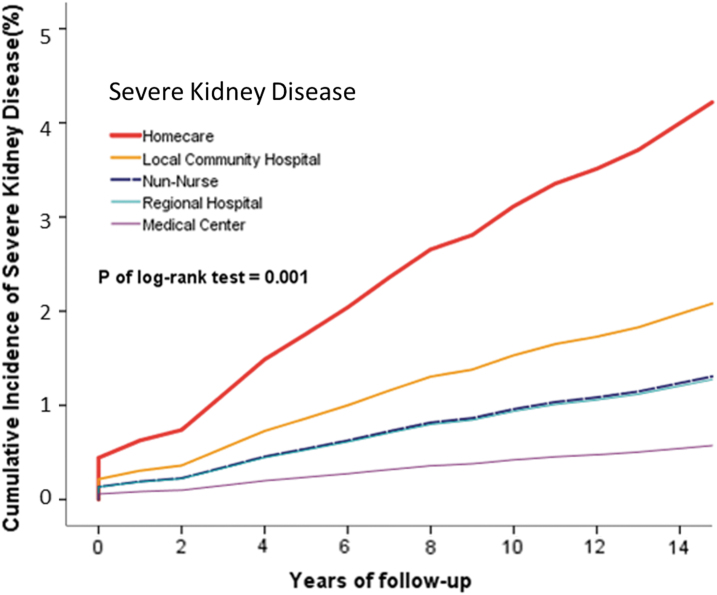
Cumulative incidence of overall severe kidney disease in homecare, medical center, regional hospital, and local community hospital nurse cohorts.

Kaplan–Meier curves were used to assess the cumulative risks of diseases in all institutional nurses, and significance was determined by using the log-rank test (Figs. 2–4).

The risk of chronic lung disease was significantly higher in homecare nurses than in non-homecare nurses (*p* = 0.018). Similar results were obtained for the risks of mild liver disease (*p* = 0.016), severe kidney disease (*p* = 0.001), intervertebral disk displacement (*p* = 0.001), and low back pain (*p* = 0.001). The risks of back pain (*p* < 0.001) and sleep disorders (*p* < 0.001) were significantly higher.

## Discussion

In this study, comparison of nurses and non-nurses showed that nurses had higher risks of chronic lung disease, connective tissue disease, ulcer disease, mild liver disease, severe liver disease, intervertebral disk displacement, low back pain, back pain, menstrual abnormalities, migraine, tuberculosis, and sleep disorders. The prevalence of diseases in nurses was higher and significantly different than those in non-nurses (*p* < 0.005).

Comparison of disease risk between homecare nurses and general hospital nurses revealed that the risk ratio was not significant for ulcerative diseases and tuberculosis in homecare nurses, but the prevalence of these diseases was still higher than that in general hospital nurses. The prevalence of chronic lung disease, mild liver disease, severe kidney disease, severe liver disease, intervertebral disk displacement, low back pain, back pain, menstrual abnormalities, migraine, and sleep disorders was significantly higher than that in general hospital nurses. Especially, the prevalence of mild liver disease was higher in homecare nurses than in medical center, regional hospital, and local community hospital nurses.

Based on the work environment, various types of nurses can be ranked in the following order based on the prevalence of diseases: homecare nurses > local community hospital nurses > regional hospital nurses > medical center nurses.

Comparison of age groups revealed that the risk of mild liver disease in homecare nurses aged 21–30 years was higher than that in the same age group. The risk of chronic lung disease, mild liver disease, intervertebral disk displacement, low back pain, back pain, menstrual abnormalities, migraine, and sleep disorders was higher in homecare nurses aged 31–40 years than in general hospital nurses. The risks of mild liver disease and disk displacement were higher in 41–50-year-old homecare nurses than in general hospital nurses. The risks of severe kidney disease, severe liver disease, intervertebral disk displacement, migraine, and sleep disorders were significantly higher in homecare nurses aged older than 51 years than in general hospital nurses.

Past research indicates that medical personnel are in direct contact with tuberculosis patients; thus, medical personnel work in a high-risk environment. Therefore, the risk of tuberculosis is higher in nurses than in the general population.^21,22^ Therefore, the risk of tuberculosis was determined in this study. The results showed that the risk of tuberculosis was significantly higher in homecare nurses, but its occurrence was still higher in homecare nurses than in general hospital nurses. Chronic lung disease is associated with smoking, and the risk of chronic lung disease is also increased with exposure to secondhand smoke.^23^ However, the prevalence of chronic lung disease was higher in homecare nurses than in non-homecare nurses. It can be inferred that the environment of homecare is a complex environment unlike the environment of a general medical institution. Homecare nurses visit the patient's home to provide care services, leading to chronic lung disease. The cause of the disease in homecare nurses is yet to be further explored.

In the definition of liver disease in this study,^24,25^ in addition to chronic hepatitis-related diseases, hepatitis B and C were also included. Nurses often need to pump blood, apply injections, and other tasks, conferring the risk of needle contact or blood contact. Accidental needlestick injury may lead to infection with hepatitis B or C through blood contact. Because of blood contact of nurses during suturing and surgery, they are at a high risk of hepatitis infection.^26,27^ In another study, it was found that the proportion of infected homecare nurses was higher than that of general hospital nurses. The reason may be that the homecare nurses were different from general hospital nurses.^28^ Some of the homecare patients are bedridden for a long time and cannot move. Homecare nurses are often exposed to vomiting, urine, feces, *etc.* of patients. Therefore, they are often exposed to body fluids and blood contact, which are risk factors for hepatitis; thus, the risk of liver disease is high for homecare nurses than for general medical nurses.

Musculoskeletal disorders such as disk displacement, low back pain, and back pain are the most common diseases among nurses because they need to help patients turn over, *etc*. The results of this study showed that the prevalence of back pain was significantly higher in homecare nurses than in general hospital nurses. In past studies, the prevalence of lower back pain was the most related to turn over patients.^29^ Homecare patients are mostly in bed for a long time, and their mobility is low. It takes more physical effort to help such patients turn over and do bed rehabilitation, which leads to an increase in the prevalence of back pain.

### Limitations

This study obtained the sample from the NHIRD of Taiwan. The data sets of the nursing staff's workload, lifestyle, socioeconomic background, and marital status were not available. In addition, clinical data were not available to verify the accuracy of ICD-9-CM codes in the NHIRD. Clinicians enter disease codes, human input may result in classification errors, and diagnostic errors made by physicians are also possible. Nurses with other medical conditions may have preferentially selected homecare occupations, but this seems unlikely.

## Conclusions

The prevalence of chronic lung disease, peptic ulcer disease, mild liver disease, severe kidney disease, severe liver disease, intervertebral disk displacement, low back pain, back pain, menstrual abnormalities, migraine, tuberculosis, and sleep disorders was higher in homecare nurses than in general hospital nurses. The correlation between different institutions and disease prevalence rates revealed that various types of nurses can be ranked in the following order based on the prevalence of the aforementioned diseases: homecare nurses > local community hospital nurses > regional hospital nurses > medical center nurses.

Heavy workloads, long working hours, workplace stress, rotating nightshifts, and inadequate coping skills may explain our epidemiological findings of higher risks for liver diseases in homecare nurses. This might help improve our health policies for homecare nurses. Education programs may be helpful in reducing the prevalence of liver diseases among homecare nurses.

## Abbreviations Used

CCI: Charlson comorbidity index
CI: confidence interval
HR: hazard ratio
ICD-9-CM: International Classification of Disease, Ninth Revision, Clinical Modification
NHIRD: National Health Insurance Research Database
PSM: propensity score matching
